# *Yalongaphaenops erwini* gen. et sp. nov., the world’s most high-altitude hypogean trechine beetle from China (Coleoptera, Carabidae, Trechinae)

**DOI:** 10.3897/zookeys.1044.62572

**Published:** 2021-06-16

**Authors:** Igor A. Belousov, Ilya I. Kabak

**Affiliations:** 1 All–Russian Institute of Plant Protection, Podbelskogo 3, 189620, St. Petersburg, Pushkin, Russia All-Russian Institute of Plant Protection St. Petersburg Russia

**Keywords:** Carabids, Sichuan, taxonomy, Trechini, troglobitic

## Abstract

A new genus and new species of carabid beetle, *Yalongaphaenops erwini***gen. et sp. nov.**, is described from mountains SW of Mianning City, Sichuan Province, China. This finding, from one side, extends the known distribution of Chinese hypogean trechines, and from another, it raises the upper limit of their vertical distribution to nearly 4000 m. Photographs of the habitus and major taxonomic characters, including the male genitalia, right mandible, and microsculpture patterns of the integument are supplied. The type locality of the new taxon is mapped. *Yalongaphaenops***gen. nov.** shows some similarities with the genus *Shiqianaphaenops* Tian, 2016 from eastern Guizhou and the genus *Boreaphaenops* Uéno, 2002 described from Hubei, China. However, the direct relationships of the new genus remain unclear. Further new discoveries of hypogean trechines in Sichuan are necessary to evaluate possible variation of major characters in allied taxa. Although *Y. erwini***sp. nov.** was collected at an elevation exceeding 3800 m a.s.l., it conforms to the upper limit of the forest zone being approximately on par with the vertical distribution of some high altitude hypogean trechine species in other parts of the globe.

## Introduction

In recent years, many troglobitic trechine beetles were discovered in southern China up to Hubei Province in the north (e.g., Tian et al. 2016; for reference data see Belousov 2017). Their taxonomic diversity and variety of body shapes have greatly influenced our knowledge about major living forms of troglobitic trechines. The overwhelming majority of these findings are restricted to a few, rather small, areas of China, mostly in Guizhou, Guanxi, and some neighboring provinces. It is worth noting that Sichuan Province, renowned for its extremely high biodiversity of carabids, is rather poorly represented in relation to known true hypogean trechines. The latter are mostly members of the genus *Sichuanotrechus* Deuve, 2005 inhabiting mountains near Beichuan in the northern part of the province (Deuve 2005; Uéno 2006a, 2008; Huang and Tian 2015). That is why the discovery of a new hypogean trechine genus, *Yalongaphaenops* gen. nov., in the southern part of Sichuan Province is of particular interest and is promising in terms of further investigations toward understanding the genesis of the hypogean fauna of China as a whole. Additionally, this new genus is the only aphaenopsoid trechine found at an altitude above 3800 m a.s.l. To date, there were only a few hypogean trechines recorded from elevations exceeding 2500 m (e.g., *Himalaphaenops* Uéno, 1980) while most specialized hypogean taxa of Trechini occur at lower altitudes. Moreover, the shift of insects from epigean habitats to the hypogean zone is supposedly promoted by increasing aridity of the environment (Faille et al. 2013) that is much more likely to take place at lower elevations.

Thus, it may be concluded that the discovery of a specialized hypogean trechine beetle at such high elevations adds one more dimension to expected insect biodiversity of south Asia.

## Materials and methods

Specimens were examined and measured using a MBS-10 stereomicroscope with an ocular-micrometer. Genitalia preparations were studied and photographed using AxioVision software version 2.6 with extended focus module and a Carl Zeiss Axio Imager M1 microscope equipped with an AxioCam MRc5 camera. Photographs of beetles were taken with a Canon 40D DSLR digital camera, using stacking and subsequently processed with Zerene stacker software version 1.04 (http://zerenesystems.com/stacker).

Under the material section, the number of specimens studied is followed by the number of the genitalia preparations given in parentheses. The holotype of the new species is housed in the collection of the Zoological Institute of the Russian Academy of Sciences (**ZIN**, St. Petersburg). The paratypes are deposited in the private collection of the authors (CBK, St. Petersburg, Russia).

The measurements taken in the present paper are the same as in our previous articles (e.g., Belousov and Kabak 2000, 2003). The body length was measured without mandibles, from the anterior margin of labrum, the width of the pronotal base at level of hind angles. The position of the anterior setiferous pore of the pronotum, the discal, preapical, and umbilicate pores on the elytra are given as percentages of the length of the pronotum and elytra correspondingly. The latter were measured from the apex of scutellum to the apex of elytra along the elytral suture. Two males are measured, including the holotype.

## Taxonomy

### Yalongaphaenops gen. nov.

Taxon classificationAnimaliaColeopteraCarabidae

577B12C9-7ED9-5AC3-AA23-EB0CCC801737

http://zoobank.org/875E7C7F-45F8-495C-82C3-68A22B9F6973

#### Type species.

*Yalongaphaenops erwini* sp. nov.

#### Diagnosis.

Among numerous aphaenopsoid and semi-aphaenopsoid genera of China, the new genus is distinct in having the following set of character states: frontal furrows incomplete; two basal tarsomeres dilated in males; rather homogeneous though very short pubescence of the body surface, two supraorbital setiferous pores on each side of head; labial suture distinct; six longer submental setae and one or two shorter setae in the same row (thus, altogether seven or eight submental setae); both labial and maxillary palpi with distinct and relatively long setae (except for the ultimate segments which are completely glabrous); only one lateral pore of pronotum (posterior one absent), elytra with two discal and one preapical setiferous pores in stria 3; two apical pores in addition to the preapical pore on apical slope; umbilicate series with pore 1 not shifted inwards, pores 1, 2 and 8 nearly attached to lateral groove, pores 4, 5, and 7 clearly removed from it and pores 3 and 6 in intermediate position; pore 5 located much closer to pore 6 than to pore 4, in other words, the median group clearly separated from the humeral group. Additionally, the shape of the tooth on the right mandible is worth noting: it is tridentate, but without isolated premolar, of triangular shape with basal denticle much longer than others. Such subtriangular shape is unusual for Chinese Trechini and, to some extent, resembles teeth of some species of the Caucasian genus *Cimmerites* Jeannel, 1928 but in the latter case, the distal denticle is completely reduced (Belousov 1998).

#### Description.

**Figure 1.**
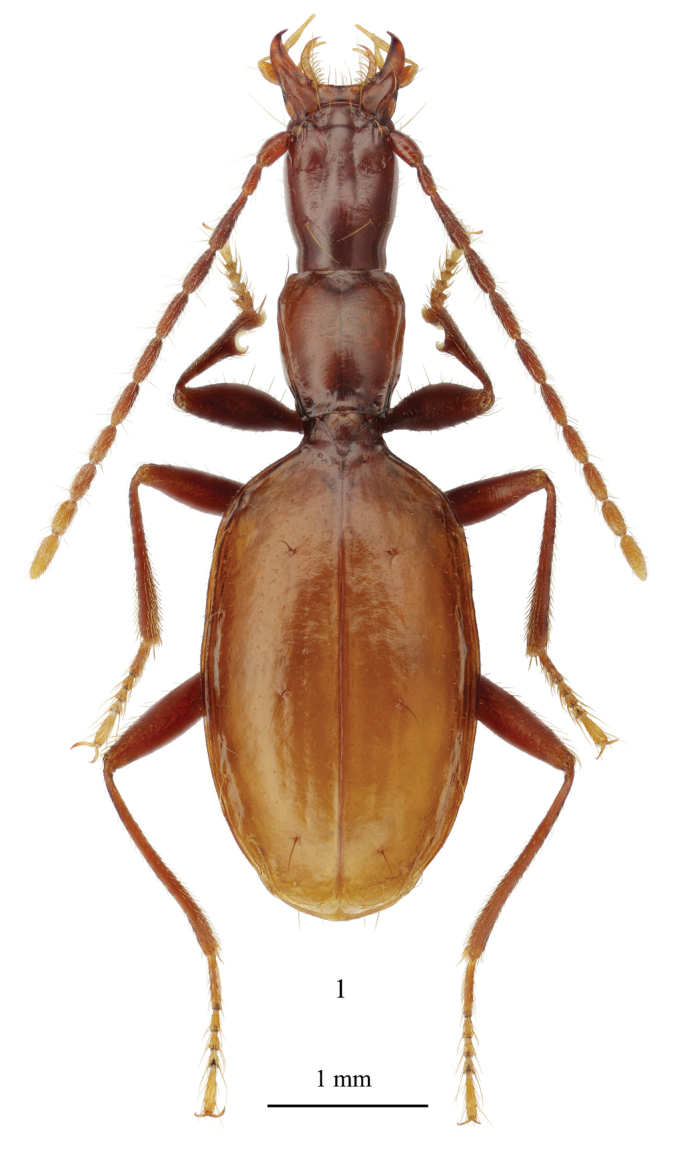
*Yalongaphaenops erwini* gen. et sp. nov., holotype, habitus.

**Figures 2–6.**
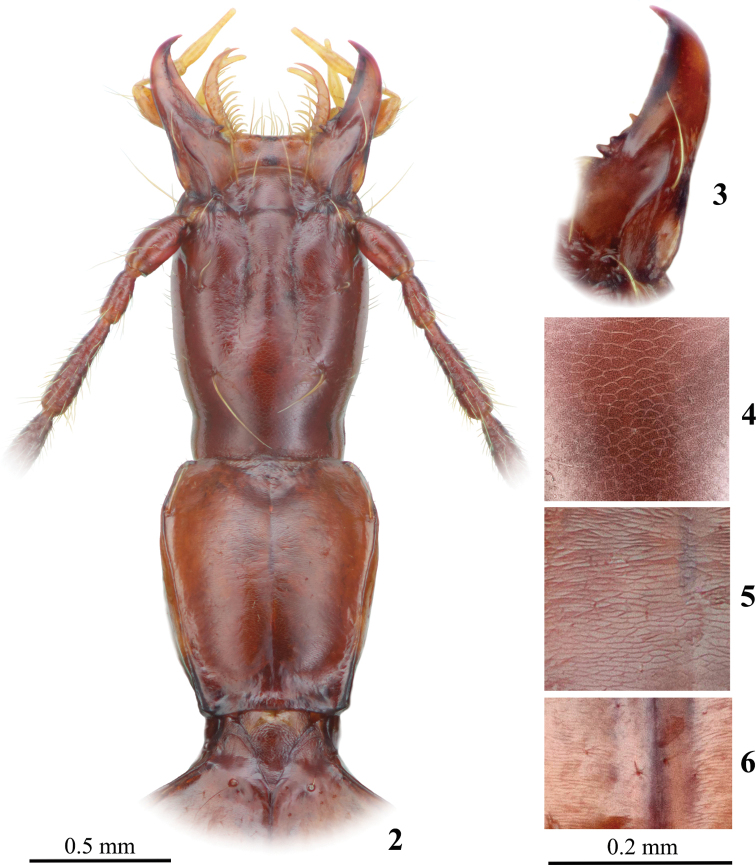
*Yalongaphaenops erwini* gen. et sp. nov. **2** forebody **3** right mandible **4–6** microsculpture: **4** occiput **5** disc of pronotum **6** disc of elytra near suture.

***Body*** medium-sized for hypogean trechines, apterous, depigmented (Fig. 1). Eyes completely reduced. All body surface sparsely, very shortly and evenly pubescent, hairs mostly suberect, more distinct and denser on anterior part of head, where they directed anteriad, nearly indistinguishable on occiput, more distinct and directed posteriad on most part of both pronotum and elytra except for their anterior portions, where hairs are erect and even directed slightly anteriad. Forebody rather narrow (Fig. 2), head nearly as wide as pronotum, elytra much wider, regularly ovate, with gently convex disk, their maximum width slightly behind mid-length, humeri distinct though rounded. Antennae and legs rather thin, moderately elongate, antennae slightly longer than elytra, third antennomere approximately twice as long as the second one. Color reddish amber, with paler testaceous elytra and three or four distal antennomeres.

**Figures 7, 8.**
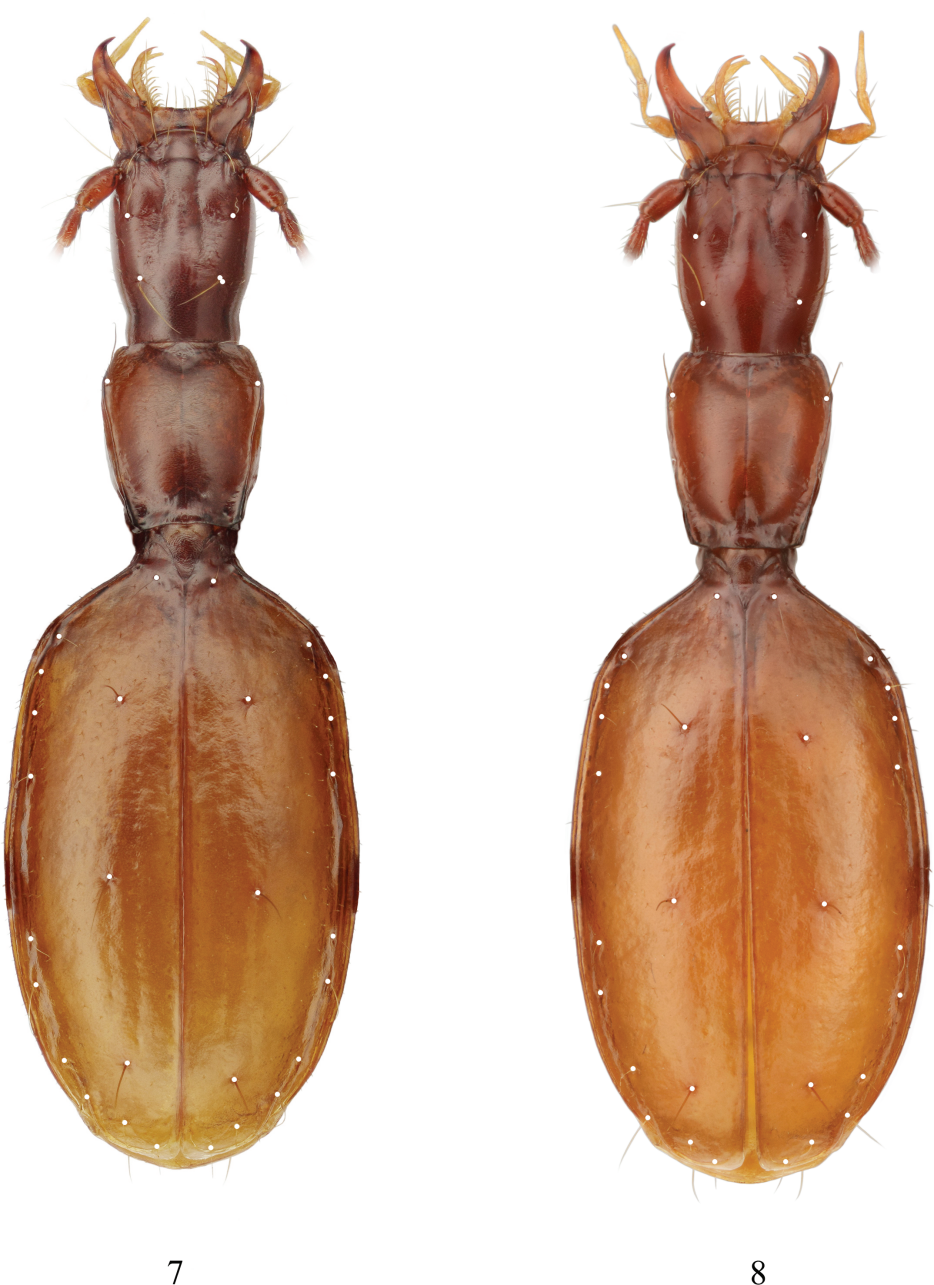
Chaetotaxy of *Yalongaphaenops erwini* gen. et sp. nov. **7** holotype **8** paratype.

***Head*** very large, subparallel-sided, barely narrower and much longer than pronotum (Fig. 2). Frontal furrows incomplete (normally not reaching posterior supraorbital seta), undulated, rather irregular and unevenly impressed, markedly approaching at level before anterior supraorbital pore. Frons moderately convex, with few shallow transverse impressions; occiput markedly convex. Tempora very long, rather flat, only slightly convex in posterior third, covered with sparse and evenly distributed setae which are slightly longer that other hairs of the body surface. Two supraorbital setae on each side of head located in lines faintly convergent posteriad (Figs 7, 8). Two clypeal setae on each side. Labrum subrectangular, markedly transverse; anterior margin nearly straight, with six setae. Mandibles short compared with head, rather stout, distinctly curved at their apical portions. Tooth on right mandible (Fig. 3) tridentate, with basal margin rather long; proximal denticle (on site of premolar) longest, not completely isolated from the median denticle which is shortest, distal denticle medium in length, removed from both proximal denticles and separated from these by a deep and rather long emargination. Thus, the tooth is largely triangular shaped, with more prominent proximal part. Tooth on left mandible bidentate. Maxillae slender, evenly arcuate. Glossa gently produced medially, with tapered apex, and a pair of very long and thick median setae and three pairs of thinner and shorter lateral setae on each side of glossa. Paraglossae markedly projected beyond anterior margin of glossa, slightly curved, with relatively long, tiny hairs on their inner margins. Labium with deeply emarginate anterior margin, markedly produced epistomes and distinct and relatively narrow labial tooth, directed mostly anteriad and blunt or cleft at apex, its ventral surface with a callosum-like convexity proximally and oblong impression in median and distal portions. Median part of labial disk convex, bearing a couple of long and closely located setae, its proximal part deeply impressed in semi-circle, with two sensorial pores, which are only marginally larger than pores of labial setae. Labial suture distinct, clearly visible even in reflected light, nearly straight in median part and sinuate laterally. 7–8 submental setae, of which the subangular setae are rather short and located just in lateral angles of submentum at level clearly before other setae, the lateral ones longest and the median 1–2 setae shortest, only marginally longer than background hairs of the underside. All pores between lateral pores arranged in more or less regular transverse row. Both maxillary and labial palpi long and slender, their apical segments glabrous, similarly shaped, long, subfusiform, not depressed, with maximum width in basal third and attenuate apically. Penultimate segment of maxillary palpi very short (only 0.60 length of ultimate segment), with narrow basal portion and markedly dilated apical part, where it is approximately as wide as ultimate segment. Second segment of maxillary palpi clearly longer than ultimate segment and much thicker than other segments; its exterior margin evenly convex except for basal extremity, inner margin sinuate. All segments of maxillary palpi, except for ultimate one, distinctly pubescent, setae rather long, becoming denser and longer on the exterior surface and toward the segment apices; the inner surface of the penultimate segment with a few long setae; that of the second segment with only one long seta located slightly closer to the segment apex and a few very short hairs. Ultimate and penultimate segments of labial palpi of subequal length, penultimate being much thicker and clearly depressed, with maximum width in apical quarter, with four setae (proximal seta located near mid-length of the segment). Upper side of head with even and sparse pubescence lacking only in posterior part of occiput, hairs rather long, directed mostly forwards, especially in anterior part of head, becoming more erect in posterior part of head. Underside pubescent, hairs suberect, rather long and sparse much similar to those on tempora.

***Pronotum*** long and narrow, much longer than wide, with maximum width in apical fifth; propleura clearly visible from above. Lateral margins of pronotum rounded in anterior part, rectilinearly convergent posteriad, slightly undulated for most of their length, without distinct sinuation before hind angles. Lateral border complete, lateral groove very narrow. Front angles barely produced. Hind angles small and obtuse, pointed apically, lateral margins near hind angles markedly reflexed upward. Basal margin rectilinear medially, obliquely truncate laterally. Basal foveae small but distinct, prebasal transverse impression well developed. Only one lateral seta located in anterior fifth of pronotum, posterior one absent (Figs 7, 8). Lateral margins ciliated, more distinctly in anterior portion of pronotum, but even here cilia more than twice shorter than cilia on lateral margins of elytra.

***Elytra*** with humeri rounded but distinguishable. Elytral disk subconvex; basal part of elytra with distinct impression. Lateral margins distinctly undulated and clearly ciliated, their humeral area smooth, not serrate. Marginal groove average in width, distinctly narrowed before humeri, widened in apical part. Discal striolation reduced, only inner striae continuous though shallow, at most, very slightly punctured. Apical recurrent striole short, nearly straight, directed anteriad, bordered by a short and thick carinula exteriorly. Parascutellar striole barely visible. Parascutellar setiferous pore present. Two long and thick discal setae and one similar preapical seta on each elytron, discal pores located in stria 3, preapical one in the apical cross of striae 2 and 3 markedly anteriad of anterior termination of apical recurrent striole (Figs 7, 8). Apical triangle of setae complete: both exterior pore and angulo-apical pore present, the former being markedly removed from apical recurrent striole and the latter attached to apical border. Umbilicate series consisting of typical fixed eight setiferous pores, divided into three groups: humeral (4 pores), median (2) and preapical (2), of which the median group is clearly shifted posteriad. Umbilicate pores 1–3 of the humeral group subequally distributed along the lateral groove and located approximately at the same distance from lateral margin while pore 4 markedly removed from pore 3 and shifted medially from lateral margin quite similarly to pore 5, which is approximately twice as distant from the lateral margin as pore 6. In the preapical group, pore 7 also shifted inward and located at level markedly before anterior termination of the apical recurrent striole. Hairs of elytral pubescence arranged mostly in one irregular longitudinal row on each interspace, all hairs suberect, becoming more adpressed posteriorly.

***Prosternal processus*** not margined, with a few rather long setae.

***Abdominal sternites*** pubescent mostly along their posterior margins, more widely in median parts of sternites, glabrous elsewhere. One pair of long paramedian setae on each abdominal sternite and one pair of setae on last visible sternite (sternite VII) in all known male specimens markedly more spaced than paramedian setae on adjacent sternites.

***Metepistenites*** clearly longer than wide.

***Legs*** long, thin, and only slightly curved. Front tibiae without deep groove on exterior surface, only depressed there, their entire surface evenly and densely pubescent. Underside of anterior femora not grooved, without tubercle in proximal part, with three longer setae along anterior margin in their basal half and several setae along posterior margin; most of the latter are located in the basal half and one seta in distal third of femora. Male protarsi with two basal segments dilated and provided with adhesive appendages beneath, inner denticle medium-sized in first tarsomere, small in second tarsomere. Fourth tarsomeres of anterior and middle legs with a small ventral apophysis surmounted by a lanceolate hyaline appendage, strongly curved apically, this appendage markedly shorter and narrower than the fifth tarsomere.

***Microsculpture*** of body surface well developed (Figs 4–6), upper side rather matt.

**Figure 9.**
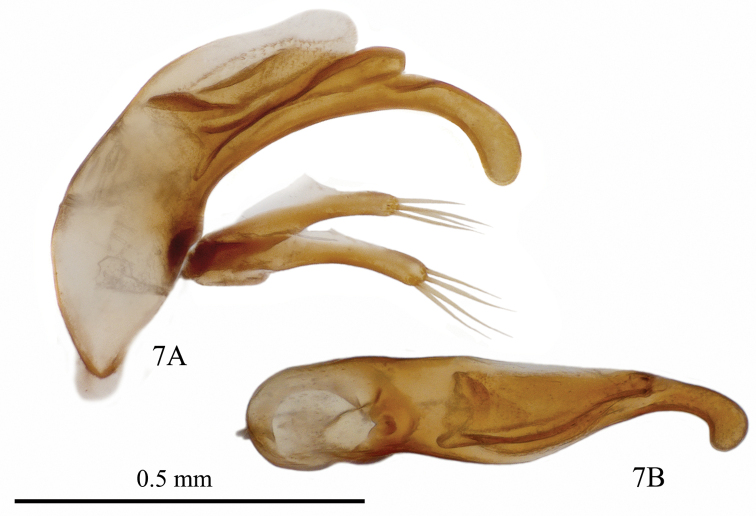
Male genitalia of *Yalongaphaenops erwini* gen. et sp. nov. **A** lateral view (holotype) **B** dorsal view (paratype).

***Male genitalia*** (Fig. 9) of peculiar shape: its distal portion markedly attenuated, curved ventrally and slightly dilated apically in lateral view. Endophallus armature rather large, well sclerotized, consisting of parietal mesh and a rather large spatulate copulatory piece concave basally and rounded apically which is located in the distal half of the median lobe (without apical lamella). Parameres of medium length, rather thin and straight, left one clearly longer, each bearing four apical setae, ventral apophysis faintly protruding.

#### Comparative notes.

Despite its medium size (body length without mandibles slightly exceeding 5 mm) and moderately elongate legs and antennae, the new genus is a rather specialized semi-aphaenopsoid trechine characterized by the hypertrophied head with frontal furrows markedly shortened posteriorly, very narrow pronotum with lateral parts of propleura visible from above and some other features commonly found in aphaenopsoid trechine beetles.

Among all blind Chinese genera of Trechini, the new genus seems to be the most similar to *Shiqianaphaenops* Tian, 2016 (Tian et al. 2016) with two currently known species: *S. majusculus* (Uéno, 1999) and *S. cursor* (Uéno, 1999). Both these species were originally treated as belonging to *Shenaphaenops* Uéno, 1999 (Uéno 1999a). The latter genus was described based on a single female specimen of *Shenaphaenops humeralis* Uéno, 1999 while *S. majusculus* and *S. cursor* were later assigned to *Shenaphaenops* (Uéno 1999b); however, he noted some important differences in characters and a gap in geographical distribution between these two species and the type species of *Shenaphaenops* (the latter was found in northwestern Guizhou while the two species of the genus *Shiqianaphaenops* inhabit caves in Shiqian County in eastern Guizhou). In 2014–2015, M. Tian and colleagues visited the type locality of *Shenaphaenops humeralis* and succeeded in collecting a male specimen of this species. The structure of the male protarsi provided one more clue character which allowed the Chinese author to isolate *S. majusculus* and *S. cursor* into a new genus, *Shiqianaphaenops* with *S. majusculus* chosen as the type species of the genus (Tian et al. 2016).

*Yalongaphaenops* gen. nov. shares with members of *Shiqianaphaenops* the following combination of characters: body pubescent; head longer than pronotum; frontal furrows completely effaced posteriorly; right mandible tridentate; labial suture clearly visible; pronotum elongate; propleura visible from above; only anterolateral seta present on pronotum (posterolateral absent); elytral margins ciliated; two discal setiferous pores and one preapical pore on each elytron; umbilicate pore 1 attached to lateral groove and not clearly shifted medially; protibiae pubescent on their anterior surface and not grooved externally; two basal segments of male protarsi dilated; male sternite VII bisetose. However, *Yalongaphaenops* gen. nov. differs from the above genus in the following characters: even and very short hairs of the upper side versus hairs much longer on head and pronotum than on elytra in *Shiqianaphaenops*; absence of the external process with ctenidium-like structure on tarsomere 1 in both sexes (a synapomorphy unique for the genus *Shiqianaphaenops*); seven-eight submental setae in *Yalongaphaenops* gen. nov. vs. nine in *Shiqianaphaenops*; two penultimate segments of the maxillary palpi densely setose in *Yalongaphaenops* gen. nov. vs. glabrous in *Shiqianaphaenops*; umbilicate pore 4 located much closer to umbilicate pore 3 than to umbilicate pore 5 in *Yalongaphaenops* gen. nov.; angulo-apical seta present in *Yalongaphaenops* gen. nov. vs. absent in *Shiqianaphaenops*; and some characters in the structure of the male genitalia: the aedeagal median lobe with lamella markedly elongated and arc-like curved ventrally (Fig. 9) and parameres with 4 apical setae in *Yalongaphaenops* gen. nov. vs. aedeagal apex straight and short and parameres with 3 apical setae in *Shiqianaphaenops*.

There are some other Chinese aphaenopsoid Trechini genera demonstrating certain affinities with *Yalongaphaenops* gen. nov.

First of all, the genus *Boreaphaenops* Uéno, 2002 is worth noting (Uéno 2002). The genus currently includes two species: *B. angustus* Uéno, 2002 from western Hubei and *B. liyuani* Tian & He, 2020 from northeastern extremity of Sichuan. The latter species is known only from one female specimen and placed into the genus by the authors with some reservations, since the two species show differences of generic importance (Tian and He 2020). For these reasons, we, first, analyze differences separately for each of these species and then make a general conclusion. *Boreaphaenops angustus* shares with *Yalongaphaenops* gen. nov. the similar size and body shape, which is elongate and rather flat, with slender appendages and rather stout mandibles. Both taxa are characterized by the following features: frontal furrows shortened posteriorly; two segments of male protarsi dilated; whole body including tempora, evenly pubescent, although hairs are sparser and shorter in *Y. erwini* gen. et sp. nov.; barrel-shaped pronotum with very narrow lateral groove and sides without distinct sinuation before obtuse hind angles, six longer submental setae, similar aggregate state of the humeral umbilicate pores which are located one after another along lateral groove of elytra. Conversely, the new genus differs from *B. angustus* in some important morphological traits: only two supraorbital setae on each side (posterior pores redoubled in *B. angustus*), absence of posterior lateral setae on pronotum; two discal setiferous pores on elytra (apart from the preapical pore) vs. three in *B. angustus*; pubescent maxillary palpi (except for ultimate segment) vs. maxillary palpi glabrous in *Boreaphaenops.* The median lobe of aedeagus is much stouter, with apical portion markedly curved ventrally vs. distinctly curved dorsally in B. angustus. Boreaphaenops liyuani differs from *Yalongaphaenops* gen. nov. in appearance: antennae, legs and mandibles are much longer (antennae nearly twice as long as elytra while approximately as long as elytra in *Yalongaphaenops*, mandibles slender, parallel-sided in their middle portion vs. gradually tapered in *Yalongaphaenops*). The pattern of the integumental pubescence is quite different: head and elytra markedly pubescent while pronotum is glabrous vs. uniform, even and very short pubescence of *Yalongaphaenops*, abdominal sternites glabrous vs. sparsely pubescent; humeri reduced vs. distinct; maxillary palpi glabrous, slender, with the penultimate segment slightly longer than the ultimate segment while maxillary palpi markedly pubescent except for the last segment, with penultimate segment clearly shorter than the ultimate one in *Yalongaphaenops*; very long penultimate segment of labial palpi vs. both ultimate segments of equal length in *Yalongaphaenops*, mentum and submentum fused as opposed to distinct labial suture in *Yalongaphaenops*, 10 vs. 6 long submental setae, glabrous lateral margins of elytra vs. ciliate in its counterpart, presence of two lateral setae on each side of pronotum, only one discal seta on elytra, two pairs of paramedian setae vs. one pair on sternites IV–VI and in many other characters of minor importance. To summarize, *Yalongaphaenops* differs from both members of *Boreaphaenops* in much shorter appendages, absence of posterior lateral setae of pronotum, different shape and chaetotaxy of maxillary palpi and other pattern of body pubescence. On the other hand, some character states such as the barrel-shaped pronotum with narrow lateral groove, the partially overlapping chaetotaxy patterns of head and elytra suggest that all three species may belong to one phyletic line.

Four species of the genus *Qianotrechus* Uéno, 2000 are described from northeastern Guizhou, one more species from southeastern Sichuan (Uéno 2000a, 2003a), and Q. (Jinfotrechus) grebennikovi Deuve from Chongqing Province (Deuve 2014). The species from Sichuan was later isolated into its own genus *Uenoaphaenops* Tian & He, 2020. *Yalongaphaenops* gen. nov. readily differs from members of this genus in the following characters: whole dorsum evenly pubescent, although hairs are very short and barely distinguishable (vs. only lateral portions of elytra pubescent in *Qianotrechus*); labial suture well developed; 6 fixed submental setae (vs. 10–15 in *Qianotrechus*); two penultimate segments of maxillary palpi setose (glabrous in *Qianotrechus*); the first umbilicate pore is not shifted medially; only two paramedian setae on abdominal sternites (vs. two pairs), and foretibiae without longitudinal groove on their exterior surface vs. nearly bicarinate in *Qianotrechus* (Uéno 2000a, 2003a). Bearing in mind that the species of *Qianotrechus* are extremely variable in some taxonomically important characters, such as the number of pronotal lateral setae and presence or absence of the preapical pore on elytra, the aforementioned characters need to be assessed for their relevancy. In this context, the difference in the number of submental setae and pubescence of the two penultimate segments of the maxillary palpi seem to be of greater importance and allow suggesting that these two genera are not closely related. On the other hand, it is worth noting that the apical portion of the aedeagal median lobe is slightly curved downward in members of *Qianotrechus*, more so in *Q. magnicollis* Uéno, 2000, recalling a peculiar shape of the aedeagal lamella in *Yalongaphaenops* gen. nov.

*Qianaphaenops* Uéno, 2000 is another aphaenopsoid genus characterized by two basal tarsomeres dilated in male protarsi and some other characters shared with *Yalongaphaenops* gen. nov. This genus is described from northeastern Guizhou and currently includes six species (Uéno 2000a; Tian and Clarke 2012; Tian et al. 2015). Members of *Qianaphaenops* are similar to *Yalongaphaenops* gen. nov. in the following characters: labial suture more or less developed; body pubescence variable enough to overlap completely the *Yalongaphaenops* gen. nov. pattern; visible abdominal sternites 3–5 with only a pair of paramedian setae; umbilicate pore 1 not clearly shifted medially in one species (*Q. longicornis* Uéno, 2000) and protibiae flat or only gently grooved on their exterior surface. On the other hand, *Qianaphaenops* differs from *Yalongaphaenops* gen. nov. in some important characters: 9–12 submental setae; presence of the posterior pronotal seta, glabrous maxillary palpi and straight apical portion of the aedeagal median lobe. Additionally, these two genera strikingly differ in the pronotal shape: the propleura are clearly visible from above in *Yalongaphaenops* gen. nov. while the lateral portions of pronotum are more developed in *Qianaphaenops* and cover completely the propleura from above.

The monotypic genus *Uenoaphaenops* Tian & He, 2020 from southeastern Sichuan is easily distinguished from *Yalongaphaenops* gen. nov. in the following set of characters: different shape of body with much wider elytra, male protarsi simple, 12 submental setae, serrate humeral margins of elytra and unusual shape of male genitalia with apical portion sharply truncate (Tian and He 2020).

Seven taxa of *Aspidaphaenops* Uéno, 2006 known from Guizhou and eastern Yunnan also share some important characters with *Yalongaphaenops*: two segments dilated in male protarsi; frontal furrows incomplete; posterior lateral seta lacking on pronotum, approximately the same number of submental setae (6–7) and similar number and position of discal setiferous pores on elytra. However, *Yalongaphaenops* gen. nov. readily differs in the following character states: integument pubescent vs. glabrous in *Aspidaphaenops*; rather short legs and antennae, the latter not reaching the apex of elytra while clearly extending beyond this level in *Aspidaphaenops*; head less elongate, with much stouter mandibles, tooth on the right mandible of different shape, with longer base and reduced distal portion (distal cusp much shorter than proximal one); mentum and submentum not fused; different structure of the pronotum with lateral groove reduced and not clearly dilated as well as propleura clearly visible from above; umbilicate pore 1 attached to the lateral groove of elytra vs. more or less shifted inward onto the elytral disc in *Aspidaphaenops*. The male genitalia differ in the apical portion curved ventrally and endophallus armature large and well sclerotized in *Yalongaphaenops* while the apical portion of the median lobe hooked dorsally and the endophallus armature poorly sclerotized in *Aspidaphaenops* (Uéno 2006b; Tian and Huang 2018; Deuve and Tian 2020).

Apart from a taxonomic approach, it seems justifiable to take a closer look at some hypogean trechines known from the geographical areas located near the discovery site of *Yalongaphaenops* gen. nov. within Sichuan and Yunnan provinces. All these areas are known as remarkable hotspots (e.g., see Deuve et al. 2016) of biodiversity of terrestrial Trechini but seem to be rather poor so far in hypogean members of the tribe.

In Sichuan, in addition to the taxa considered earlier, there are only a few true specialized hypogean genera including one endemic genus, *Sichuanotrechus* Deuve, 2005, with five species known so far from the northern part of the province (Deuve 2005; Uéno 2006a, 2008; Huang and Tian 2015). Members of this genus can be easily distinguished from *Yalongaphaenops* gen. nov. by: frontal furrows more or less complete; pronotum with lateral portions well developed, propleura not visible from above, and upper side mostly glabrous.

Quite recently, one more genus *Chu* Tian & He, 2020 with a single species, *Ch. pheggomisetoides* Tian & He, 2020, was described from the northeastern part of the province. It is an isolated genus, readily differing from *Yalongaphaenops* in peculiarly shaped pronotum with large and acute hind angles, 8 submental setae, umbilicate pore 1 clearly shifted inward and unusual elongate shape of the median lobe (Tian and He 2020).

Apart from the above genera, there are only a few anophthalmoid taxa such as *Duvalioblemus* Deuve, 1995 including one species which is known only from caves so far; Duvalioblemus (Shublemus) liyuani Deuve, He & Tian, 2020 (Deuve et al. 2020).

Yunnan Province is also rather poor in blind subterranean trechines. Apart from genera discussed above, there are only a few other taxa known so far: *Dianotrechus* Tian, 2016 with one anophthalmic species; a few species of the genus *Guizhaphaenops* Vigna Tagliani, 1997; the monotypic *Junaphaenops* Uéno, 1997 from eastern Yunnan; two troglobitic species of the genus *Shilinotrechus* Uéno, 2003; and *Yunotrechus* Tian & Huang, 2014 with a single troglobitic species.

*Yalongaphaenops* gen. nov. differs from the only species known of *Dianotrechus*, *D. gueorguievi* Tian, 2016, first of all, in its semi-aphenopsoid appearance (vs. anophthalmoid in *Dianotrechus*): much larger size; longer appendages; frontal furrows incomplete, effaced posteriorly; pronotum elongate, not quadrate; lateral portions of propleura visible from above; prehumeral margins of elytra oblique (perpendicular, even forming a re-entrant angle in the counterpart); pubescence uniform and rather short of the upper side (vs. only a few fine hairs on pronotum coupled with rather long hairs evenly distributed on elytra in *Dianotrechus*); maxillary palpi clearly setose (only two tiny setae near the apex of the penultimate antennomere in *Dianotrechus*); submentum not fused with mentum; only one (anterolateral) setiferous pore present on the pronotum; umbilicate pore 5 is not shifted anteriad (Tian et al. 2016).

The only known species of the genus *Junaphaenops*, *J. tumidipennis* Uéno, 1997, is rather similar externally to *Yalongaphaenops* gen. nov.: both taxa are of approximately the same size and have the similar pubescence and appearance except for *Junaphaenops* is more robust, especially as far as the shape of elytra is concerned. However these two taxa differ in many important characters: umbilicate pore 1 is markedly shifted inward and backward and located at level behind umbilicate pore 2 in *Junaphaenops* while all humeral umbilicate pores are arranged in a regular row in *Yalongaphaenops* gen. nov.; only one basal segment dilated of male protarsi in *Junaphaenops* vs. two basal segments dilated in the counterpart; the penultimate segment of maxillary palpi glabrous, with only a couple of tiny hairs near the apex in *Junaphaenops* while it is clearly multisetose in *Yalongaphaenops* gen. nov.; two lateral setiferous pores on each side of pronotum in *Junaphaenops* vs. posterior lateral setiferous pore absent in *Yalongaphaenops* gen. nov. (Uéno 1997).

The genus *Shilinotrechus* Uéno, 2003 (Uéno 2003b) currently includes two species of strange appearance, very unusual for hypogean trechines. The type species of the genus, *Sh. fusiformis* Uéno, 2003, was described from Shilin Xian, eastern Yunnan, while the second species, *Sh. intricatus* Huang & Tian, 2015 – from Kunming vicinity, also Yunnan Province. Members of this genus easily differ from *Yalongaphaenops* gen. nov. in the trapezoid pronotum with wide lateral groove and anterior angles clearly produced; two lateral setae on each side of pronotum; indistinct scutellum and elytra very broad at basal half, with margins slightly serrate in basal portion; only one dilated segment in male prorarsi; 10 submental setae, and some other characters of minor importance.

The only species known of *Yunotrechus*, *Y. diannanensis* Tian & Huang, 2014, readily differs from *Yalongaphaenops* gen. nov. in its anophthalmoid appearance; mostly glabrous surface of the upper side; basal segments not dilated of male protarsi; mentum and submentum completely fused and some other characters (Tian and Huang 2014).

The genus *Guizhaphaenops* Vigna Taglianti, 1997 includes now two subgenera, of which one species of the nominate subgenus and all three taxa of the subgenus Semiaphaenops Deuve, 2000 were found in Yunnan. From all these taxa, *Yalongaphaenops* gen. nov. differs in two basal segments of foretarsi dilated in male; the pronotum much more elongate, with maximum width markedly before mid-length and propleura visible from above; umbilicate pore 1 attached to lateral gutter of elytra, and more or less even pubescence of pronotum and elytra (lateral areas of elytra more distinctly pubescent in *Guizhaphaenops*), etc. (Vigna Taglianti 1997; Deuve 2000; Uéno 2000b).

Finally, *Yalongaphaenops* gen. nov. should be compared to *Himalaphaenops* Uéno, 1980 with one known species, *H. nishikawai* Uéno, 1980, which is the only hypogean trechine species found so far at an elevation exceeding 2700 m (Uéno 1980). *Yalongaphaenops* gen. nov. easily differs from this genus in the following set of characters: body surface shortly pubescent vs. glabrous in *Himalaphaenops*; foretibiae densely pubescent on anterior surface, without distinct groove on external surface vs. sharply grooved and glabrous in *Himalaphaenops*; penultimate segment of maxillary palpi multisetose vs. glabrous; shape of pronotum much more evolved, with propleura visible from above and sides without deep emargination before hind angles; posterior lateral seta of pronotum missing; umbilicate pore 1 not shifted inward and backward onto disc of elytra and apical portion of aedeagus attenuated, dilated and curved ventrally vs. the oblique button-like apex in *Himalaphaenops*.

To summarize, *Yalongaphaenops* gen. nov. does not seem to show direct relationships with any other hypogean genera of East Asia, and can be easily identified based on the two proximal segments dilated in male protarsi; penultimate segment of maxillary palpi clearly setose; and elytra with umbilicate pore 1 attached to lateral groove, the preapical pore present and two discal setiferous pores in stria 3.

#### Etymology.

The genus epithet derives from the genus *Aphaenops* Bonvouloir, 1862 and the river Yalong.

### Yalongaphaenops erwini sp. nov.

Taxon classificationAnimaliaColeopteraCarabidae

2EF397CF-5119-5E49-89F4-714A1871480E

http://zoobank.org/576E8311-590C-418E-B0EC-E709E777F8B9

Figures 1 , 2–6 , 7, 8 , , 9

#### Material examined.

***Holotype***: China • 1(1) male, with label data “China, Sichuan Province, SW Mianning Town; 28°15'27"N, 101°43'51"E; H = 3805 m a.s.l.; 11.07.2011; Belousov & Kabak leg.” (ZIN).

***Paratypes*.** 2(1) males (one male teneral) with same data as holotype (CBK).

#### Diagnosis.

Since *Y. erwini* gen. et sp. nov. is the only known species of the genus, and it is difficult, for the moment, to give a consistent species diagnosis different from that of the genus. However, the combination of the highly specialized, barrel-like shape of pronotum with rather short appendages (antennae only marginally longer than elytra), and only one pair of pronotal setae may be of major importance for species identification.

#### Description.

***Body*** length 5.15–5.21 mm. Forebody narrow, head slightly narrower and much longer than pronotum, elytra rather large, with distinct humeri and subconvex disk (Fig. 1). Legs and antennae thin and rather long. Amber-reddish with paler testaceous elytra and three or four distal antennomeres; 4–6 basal antennomeres rather dark. Legs reddish amber, with vaguely obscured femora; first tarsomere of all legs noticeably darker than the ultimate one, which is rather pale yellowish. Microsculpture of dorsum distinct, surface matte. Mesh pattern of microsculpture strongly varying on different parts of head from large shallow isodiametric cells on convex parts of frons and occiput (Fig. 4), to markedly transverse coarse cells in impressed portions of head. Pronotum with rather regular microsculpture consisting of clearly transverse meshes (Fig. 5). Microsculpture of elytra (Fig. 6) more irregular, consisting of shallow very transverse cells, strongly varying in shape depending on their location in respect to striae and setiferous pores. Microsculpture of underside rather shallow, consisting of transverse meshes. Proportions of the body are given in Table 1.

**Table 1.** Morphometric characteristics of *Yalongaphaenops erwini* gen. et sp. nov. Abbreviations: AL – length of antennae; BH – height of body, i.e., maximum thickness of abdomen with elytra in lateral view; EL – length of elytra; EW – width of elytra; HW – width of head; L2 – length of antennomere 2; L3 – length of antennomere 3; PA – width of pronotum between anterior angles; PB – width of pronotum at base; PL – length of pronotum; PW – maximum width of pronotum; TaL – length of hind tarsus; TiL – length of hind tibia; W3 – width of antennomere 3; PSa – distance from anterior margin of pronotum to anterior lateral seta of pronotum; D1 – distance from the scutellum apex to level of anterior discal setiferous pore of the left elytron; D2 – distance from the scutellum apex to the level of posterior discal setiferous pore of the left elytron; D3 – distance from the scutellum apex to the level of preapical setiferous pore of the left elytron; U1–8 – distance from the scutellum apex to the level of the corresponding umbilicate pore of the left elytron.

Index	Range	Index	Range	Index	Range
PW/HW	1.08–1.09	L3/L2	2.01–2.11	(U1/EL) × 100	11.9–13.0%
EW/HW	2.30–2.36	EL/BH	2.70–2.82	(U2/EL) × 100	17.0–18.3%
PL/PW	1.18	TiL/TaL	1.42–1.51	(U3/EL) × 100	21.4–24.0%
(PSa/PL) × 100	19.6–22.8%	EL/EW	1.64–1.66	(U4/EL) × 100	31.6–34.6%
PW/PB	1.47–1.50	EW/PW	2.13–2.16	(U5/EL) × 100	60.3–62.0%
PA/PB	1.05–1.12	(D1/EL) × 100	22.1–23.4	(U6/EL) × 100	69.1–70.2%
AL/EL	1.14–1.15	(D2/EL) × 100	51.9–52.3	(U7/EL) × 100	82.2–83.2%
L3/W3	3.65–4.10	(D3/EL) × 100	83.2–84.2	(U8/EL) × 100	90.0–90.1%

***Head*** (Fig. 2) very large, subparallel-sided, with maximum width markedly before mid-length, gradually narrowed posteriad. Frontal furrows incomplete, reaching approximately level of posterior supraorbital setae, unevenly arcuate and impressed, becoming deeper near clypeal and parietal impressions, the latter distinct. Usually two supraorbital pores on each side of head, in the holotype: two posterior supraorbital pores on the right side (thus, three pores on one side and two on the other); anterior supraorbital pore moderately foveolate, posterior one slightly so, located in lines barely convergent posteriorly (Figs 7, 8). Labrum subrectangular, distinctly transverse, its anterior margin nearly straight (lateral lobes barely produced), six setae. Mandibles rather short compared to head, more distinctly curved apically, tooth on the right one with rather long base, with proximal denticle much larger than others (Fig. 3). Maxillary palpi long and slender, penultimate segment more than 1.5 times longer than two adjacent segments. Antennae long. The second antennomere is shortest, the third one the longest; antennomeres 4–10 becoming gradually shorter, beginning with antennomere 8 not longer than the first segment (scapus); antennomere 11 approximately as long as each of antennomeres 4 and 5 and clearly longer than antennomere 10. For ratios of second and third antennomeres see Table 1.

***Pronotum*** (Fig. 2) clearly longer than wide, moderately constricted to base, with lateral margins shortly rounded in apical fifth, then rectilinearly convergent posteriad, though more or less regularly undulated for most of their length, without distinct sinuation before hind angles. Basal margin rectilinear or slightly concave medially, obliquely truncate laterally. Front angles broadly rounded, barely produced beyond anterior margin, the latter nearly straight or slightly concave medially. Hind angles of pronotum not large but distinct, pointed apically, directed outwards and occasionally posteriad. Both lateral groove and lateral border continuous, becoming very subtle behind anterior lateral seta and slightly widened again toward hind angles (in posterior 4/10 of the length). Anterior lateral seta located at broadest point of pronotum, approximately in anterior fifth of length. Posterior seta absent (Figs 7, 8). Prebasal transverse impression rather shallow, especially so in median part, more sharply engraved in basal foveae which are rather small, clearly outlined and well impressed. Base of pronotum smooth, with a few shallow and irregular wrinkles. Apical transverse impression vague and shallow. Median line distinct, becoming much deeper at level of both apical and prebasal transverse impressions.

***Elytra*** not large, but rather wide, with well-developed humeri and broadly rounded apex, their maximum width slightly behind mid-length. Preapical sinuation distinct. Lateral margins markedly reflexed, marginal groove rather wide. Basal slope of elytra with only first elytral stria well impressed. Parascutellar striole rudimentary, very short. Discal striation reduced: only striae 1 and 2 rather complete, stria 3 only partially traceable, usually more or less distinct between two discal setiferous pores, clearly extending beyond level of the second discal setiferous pores in one specimen, smooth or vaguely punctate. All interspaces flat. Anterior discal setiferous pore located at level slightly behind umbilicate pore 3 while the second one clearly before umbilicate pore 5 (Figs 7, 8). Preapical pore located in the apical cross at level near or behind umbilicate pore 7. Exterior pore of apical triangle markedly shifted inward and forward, being located at level near middle of the apical striole and equidistant between the latter and angulo-apical pore which is attached to marginal border and subequally distant from elytral suture and apical margin. Inner side of the apical triangle (formed by three apical pores including the preapical pore as a front summit) markedly convergent to the elytral suture posteriad. Apical slope with striae reduced: only stria 1 traceable though shallow, sometimes stria 2 extended posteriad beyond level of the preapical pore. Apical recurrent striole short, nearly straight, directed anteriad, without clear connection with any of discal striae. Umbilicate pore 1 located approximately at level of humerus. For detailed data on position of umbilicate pores see Table 1.

***Metepisternite*** clearly longer than wide. Abdominal ventrites shortly pubescent, each with two long paramedian setae (one on each side) and occasionally with still few shorter setae.

***Legs*** long and thin; front tibiae barely S-curved; middle tibiae nearly straight, only slightly curved near femoral joint; hind tibiae distinctly S-curved; all tibiae densely pubescent in distal third, middle and hind tibiae on inner surface, front tibiae on anterior and exterior surfaces. First tarsomere of posterior legs very long, not shorter than three following segments combined. Tarsomere 4 on pro- and mesotarsi possess a well-developed ventral tubercle furnished with hyaline appendage, the latter is narrow, curled apically, not reaching the apex of tarsomere 5. Male protarsi with two basal segments dilated: first tarsomere markedly elongate, with inner tooth not large but distinct, second tarsomere subquadrate, approximately as long as wide, with very small inner tooth. Both dilated basal segments of male protarsi with small and pairwise adhesive appendages beneath which are clearly shifted inwards and distally in first tarsomere and inwards and proximally in the second.

***Aedeagus*** (Fig. 9) not large (especially compared with the body size), markedly curved, with elongate apical portion, curved ventrally. Sagittal crest small and poorly sclerotized. Basal orifice moderately emarginate. Endophallus armature well developed, heavily sclerotized, with a distinct mesh. Parameres slender, with rather narrow distal portions, left one slightly longer, with a small ventral apophysis, each paramere bearing 4 apical setae.

**Female**: unknown.

#### Distribution.

**Map 1.**
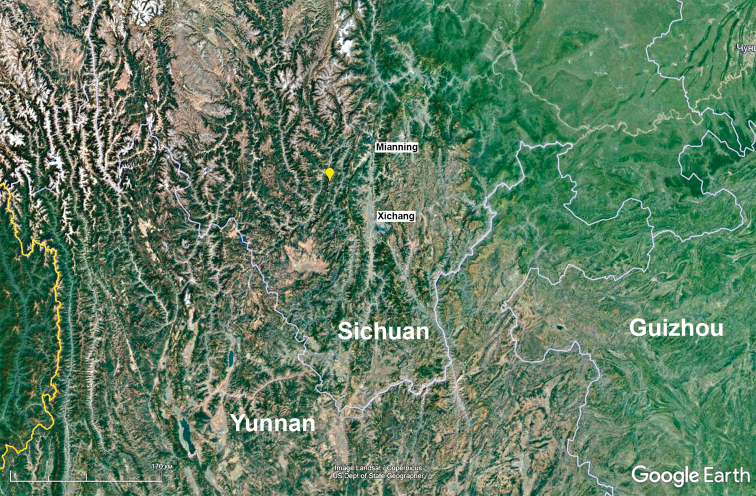
Type locality of *Yalongaphaenops erwini* gen. et sp. nov. (yellow pushpin).

China, Sichuan Province, mountains on the right bank of the Yalongjiang River, in its largest bend located SW of Mianning City (Map 1).

#### Bionomics.

**Figure 10.**
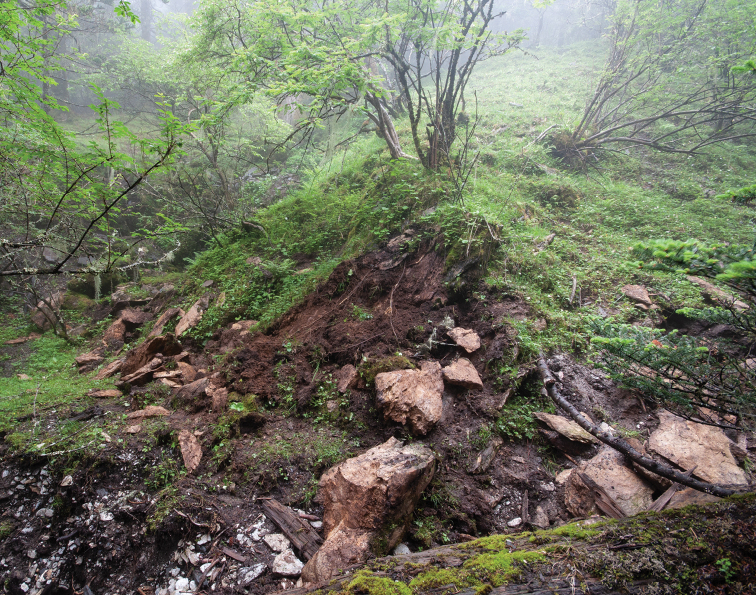
Typical biotope of *Yalongaphaenops erwini* gen. et sp. nov.

The species was found under deeply buried boulders and by splitting rocks near the entrance to a small cave with a creek flowing into it, in the upper forest zone (mostly coniferous tree species and deciduous shrubs) just below the treeline at an elevation of 3805 m a.s.l. (Fig. 10). *Yalongaphaenops erwini* gen. et sp. nov. is the most high-altitude specialized semi-aphaenopsoid trechine beetle known so far. The area where it was collected shows many signs of carbonate lithology. Apart from the above species, some other depigmented carabids with partially reduced eyes were found here, including members of the genus *Pterostichus* Bonelli, 1810 and one new cryptophilic species of *Agonotrechus* Jeannel, 1923. Based on both its appearance and the habitat, *Yalongaphaenops erwini* sp. nov. is a true hypogean species. In this respect, we prefer to follow S.-I. Uéno (1995) in his general approach to hypogean insects as well as M. Giachino and D. Vailati (2017) in avoiding, where possible, speleocentric terms like “troglobitic”. It is particularly the case of the species in question, which was collected rather close to the soil surface but at a high altitude and under humid, low-temperature conditions of the monsoon season. From the ecological standpoint, this finding is in line with our previous observations on the hypogean carabids in the Caucasus.

#### Notes.

**Map 2.**
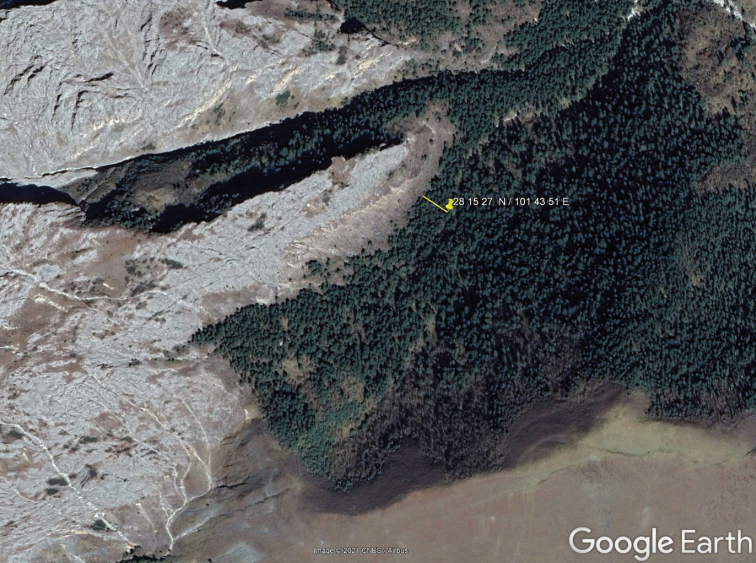
Relief map of the area nearby the type locality of *Y. erwini* gen. et sp. nov. The yellow line shows the closest distance to the upper forest limit

It was quite unexpected to find a highly specialized hypogean semi-aphaenopsoid species at an elevation exceeding 3800 m. Usually hypogean species are much more common at lower elevations, for example, the sweet spot of their biodiversity in the Caucasus is confined between 700 and 1300 m (Belousov 1998). Much more seldomly, some hypogean species can be found there at lower or higher elevations. In the latter case, there are some taxa known so far exclusively from high elevations: the genus *Taniatrechus* Belousov & Dolzhanski, 1994 (entrances to the caves populated by this species are located clearly above treeline), *Meganophthalmus kravetzi* Komarov, 1993, Nannotrechus (Abasorites) gracilipes Belousov, 1998 and eastern species of the subgenus Archinannotrechus Belousov, 1998 of the genus *Nannotrechus* Winkler, 1926; all these taxa were found either in the upper forest belt not far from the treeline or even slightly above (Belousov 1998). Likewise, in China, the highest biodiversity of hypogean Trechini is recorded for lower mountains of southern China (e.g., Tian et al. 2016). In this ecological context, the discovery of *Y. erwini* gen. et sp. nov. not far from treeline is consistent with the above data, except that the upper limit of the forest zone is located in the Tibetan plateau, including the Hengduan mountains as its eastern periphery, much higher than elsewhere in the Northern Hemisphere, with an impressive record of 4900 m documented for the southern part of Tibet (Miehe et al. 2007). In the area where *Y. erwini* gen. et sp. nov. was collected, the upper limit of the forest varies from 3600 to 4100 m a.s.l. (Map 2). Given the potential impact of livestock grazing and based on the uppermost tree stands forming part of the treeline ecotone (Miehe et al. 2014), the treeline here is supposed to be equal to approximately 4100 m a.s.l.. However, the type locality of *Y. erwini* gen. et sp. nov. is situated only 50 m from (Map 2) and 20–30 m below the adjacent timberline and the latter seems to be naturally bordered by a steep rocky ridge completely deprived of tree stands.

**Figure 11.**
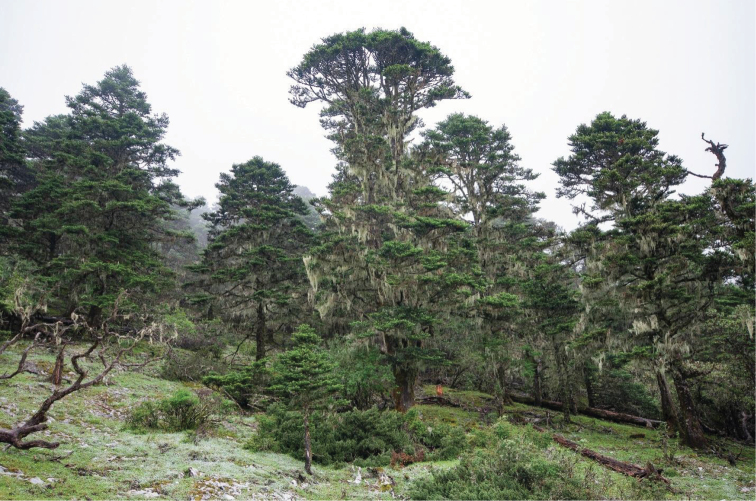
Fir forest near collecting site of *Yalongaphaenops erwini* gen. et sp. nov.

On the other hand, the treeline does not directly impact the distribution of soil-dwelling, let alone hypogean carabid species. This is particularly true for the above-mentioned highest treelines composed mostly of *Juniperus* species (Miehe et al. 2007) in southern Tibet. These tree species do not provide shade enough to harbor communities of mesophile carabids. In this regard, the fir forests and even broadleaved *Rhododendron* shrubs are much more important in creating a suitable environment for many epigean carabid species. This influence is much less evident in relation to hypogean species, but it seems very likely that the latter occur closer to the soil surface under more humid conditions. Such types of forests depend mostly on the amount of precipitation and, in the Hengduan mountains, are typical to the east-exposed slopes which are more influenced by the monsoon. That is just the case of the area under consideration. Interestingly, the tall fir forest in the immediate vicinity of the type locality of *Y. erwini* gen. et sp. nov. (Fig. 11) disintegrates very rapidly (over vertical limits of a few dozen meters) into small individual trees just as noted by Miehe et al. (2007) for the inner Himalayas of Bhutan.

However, tall fir trees near the type locality of *Y. erwini* gen. et sp. nov. met landscapes of the alpine zone in a sharp line and were directly exposed to clouds coming from open areas, catching moisture from the air as do screes and stony slopes in high mountains. The drip of the trees was particularly intensive in a narrow band along boundary of the forest resulting in a kind of permanent rain. The analogy with stony habitats was unexpectedly confirmed by the spatial distribution of some petrophilous and riparian carabids including members of *Nebria* Latreille, 1802 and the highly specialized *Leistus* Frölich, 1799 which were abundant on tree trunks in this band and completely missing elsewhere, even in stony biotopes and in the valley of a small creek.

In a sense, both the high-altitude occurrence of *Y. erwini* gen. et sp. nov. and its ecosystem association could be interpreted in terms of the lee and mass elevation effects greatly influencing the vertical distribution of carabid beetles throughout the world (Liebherr and Zimmerman 2000; Schmidt et al. 2017).

The discovery of *Y. erwini* gen. et sp. nov. significantly expands both the area and the altitudinal range where subterranean trechines are likely to be found, and suggests more field work is needed to explore their diversity in the entire Tibetan region within a wide range of altitudes.

#### Etymology.

The species is named after Terry Erwin, the great American entomologist in recognition of his invaluable contribution to our knowledge of insect biodiversity as well as taxonomy, ecology, and evolution of carabid beetles.

## Supplementary Material

XML Treatment for Yalongaphaenops

XML Treatment for Yalongaphaenops erwini

## Supplementary materials

Supplementary material 1 Table S1 Data type geographic coordinates Explanation note Geographic coordinates of the type locality of *Yalongaphaenops erwini* gen. et spec. nov. https://binary.pensoft.net/file/553392This dataset is made available under the Open Database License (http://opendatacommons.org/licenses/odbl/1.0/). The Open Database License (ODbL) is a license agreement intended to allow users to freely share, modify, and use this Dataset while maintaining this same freedom for others, provided that the original source and author(s) are credited.Igor A. Belousov, Ilya I. Kabak
